# “Skin-Core-Skin” Structure of Polymer Crystallization Investigated by Multiscale Simulation

**DOI:** 10.3390/ma11040610

**Published:** 2018-04-16

**Authors:** Chunlei Ruan

**Affiliations:** School of Mathematics and Statistics, Henan University of Science and Technology, Luoyang 471023, China; ruanchunlei@haust.edu.cn

**Keywords:** “skin-core-skin” structure, flow-induced crystallization, multiscale simulation, crystal morphology

## Abstract

“Skin-core-skin” structure is a typical crystal morphology in injection products. Previous numerical works have rarely focused on crystal evolution; rather, they have mostly been based on the prediction of temperature distribution or crystallization kinetics. The aim of this work was to achieve the “skin-core-skin” structure and investigate the role of external flow and temperature fields on crystal morphology. Therefore, the multiscale algorithm was extended to the simulation of polymer crystallization in a pipe flow. The multiscale algorithm contains two parts: a collocated finite volume method at the macroscopic level and a morphological Monte Carlo method at the microscopic level. The SIMPLE (semi-implicit method for pressure linked equations) algorithm was used to calculate the polymeric model at the macroscopic level, while the Monte Carlo method with stochastic birth-growth process of spherulites and shish-kebabs was used at the microscopic level. Results show that our algorithm is valid to predict “skin-core-skin” structure, and the initial melt temperature and the maximum velocity of melt at the inlet mainly affects the morphology of shish-kebabs.

## 1. Introduction

Currently, semicrystalline polymers are widely used in industry [1]. Usually, such polymers should be processed to become useful products. Common processing techniques include injection molding, extrusion molding, blow molding, and so others. Among these, injection molding is the most widely used. It involves a high-speed flow field and complex temperature field. These processing conditions are key factors in determining the microstructure of the products (crystal types, crystal orientation, etc.). On the other hand, the mechanical properties of the products (strength, modulus, etc.) are strongly dependent on these microstructures. Therefore, it is of great significance to control the microstructures by precisely applying external flow and temperature fields to improve the performance of the products.

**Figure 1:**
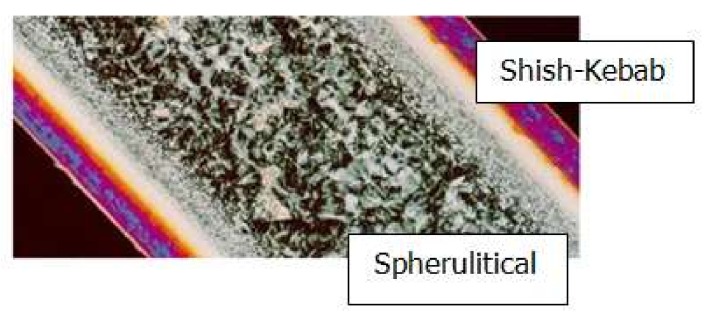
“Skin-core-skin” structure in an injection polymer product [2].

Experimental results show that the crystalline structure in the final injection product takes on a typical “skin-core-skin” structure: the shish-kebab structure with high orientation appears in the skin layer, and the spherulitical structure with essentially no preferred orientation appears in the core layer [2]. Figure 1 shows the cross-section of the shish-kebab structure and spherulitical structure of an injection product [2]. It has been reported that in the skin layer, because of the high shear stress and shear strain, the extended polymer chains lead to extended chain crystals and, ultimately, shish-kebab structures. In the core layer, because of the absence of shear, the random polymer chains lead to lamellar, chain-folded crystals and, finally, spherulites. Hence, the shish-kebab structure is related to flow-induced crystallization, and the spherulitical structure is related to quiescent crystallization [2,3,4]. Compared with the spherulitical structure, the shish-kebab structure improves the performance of products in tensile strength, tensile elastic modulus in stress direction, but reduces the performance of products in impact strength in the direction perpendicular to the stress [5]. Therefore, it is important to predict the spherulitical structure and shish-kebab structure precisely.

To date, several numerical works have reported capturing the evolution of the spherulitical structure in quiescent crystallization. For example, Raabe [6] and Spina et al. [7] used the cell automaton method to simulate spherulite growth in polymer crystallization; Ketdee [8] presented Monte Carlo simulations to predict the kinetics and morphology of isothermal polymer crystallization; Ruan et al. [9,10] applied the pixel coloring method to capture the spherulite evolution in isothermal and non-isothermal polymer crystallization; and Liu et al. [11,12] constructed a level set method to capture the spherulite development during the polymer cooling stage.

Compared with the spherulitical structure in quiescent crystallization, models and methods for the determination of shish-kebab structure in flow-induced crystallization are rare. Eder [13] considered shish-kebabs as growing cylinders and obtained a series of differential equations by using the Schneider rate equation [14]. Zuidema et al. [15] thought that recoverable strain was the driving force for the nucleation of shish-kebabs and modified the Eder model. Zinet et al. [4,16] and Mu et al. [17] used a modified Schneider rate equation to describe the growth of thermally and flow-induced nuclei. Guo et al. [18,19] introduced a molecular deformation factor, which distinguished spherulites and shish-kebabs by comparing the molecular deformation factor with the critical one. Wang et al. [20] presented a phase field method to simulate the shish-kebab structure in simple shear and temperature fields. Although the above works are based on crystal morphology, crystallization kinetics models are also needed. Crystallization kinetics models, such as the Avrami model, are often reported with lower accuracy at the later stage of polymer crystallization. Furthermore, these works did not construct a method to reveal the details of shish-kebabs. Recently, Ruan et al. [21,22,23] presented a Monte Carlo method to simulate spherulites and shish-kebabs in a parametrical study, with simple shear flow and Couette flow. They obtained detailed crystal morphology and reliable crystallization kinetics without using a kinetics model.

In this study, we extended the multiscale method to simulate the “skin-core-skin” structure of the polymer crystallization in a pipe flow, which is treated as a simplification of the injection processing. Unlike our previous work of Couette flow [22], the conservation at the macroscopic level in pipe flow is more complicated. Therefore, the SIMPLE (semi-implicit method for pressure linked equations) algorithm on collocated coarse grid was used to calculate the flow and temperature fields at the macroscopic level. Rhie–Chow-type interpolation was introduced to overcome the pressure-velocity decoupling. The morphological Monte Carlo method on fine grid was used to capture the crystal growth fronts and compute the relative crystallinity. Effects of external flow and temperature fields, (e.g., temperature cooling rate of the mold, initial melt temperature, maximum velocity of the melt in inlet) on the crystal morphology were investigated and are herein discussed.

## 2. Mathematical Model

**Figure 2:**
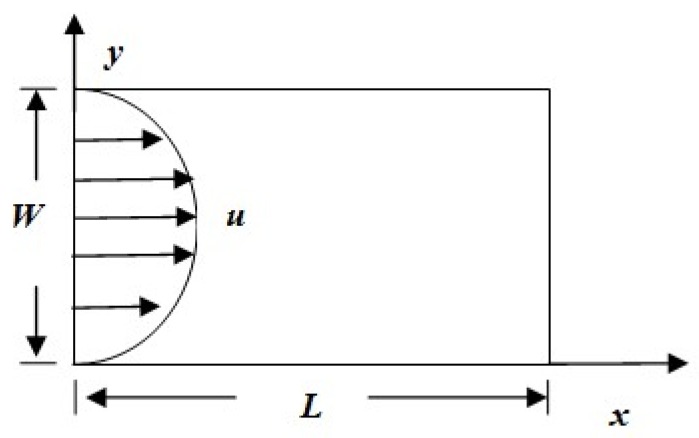
The pipe model for simulation.

In injection molding, polymer melts are injected into a mold to form different products. A changing flow domain is more suitable. Some software, such as C-mold and Moldflow, can address all stages of injection. In this work, we assumed the mold is a pipe, which is shown in Figure 2. Actually, the mold is supposed to have a thin-wall thickness in the *y* direction, which means the length in the *x* direction is substantially larger than the length in the *y* direction (*L* >> *W*). Our aim was to simulate the crystallization during and after shear treatment, which refer to the injection and cooling stages, respectively. We assumed the melt experiences shear effects with a parabolic velocity in the inlet that lasts for shear time *t_s_* (injection stage). We also assumed that after the shearing flow, the mold has experienced a large temperature change (cooling stage). Therefore, the mathematical model at the macroscopic level should be divided into two cases.

### 2.1. Conservation Equations at the Macroscopic Level

We assumed the polymer melt is a non-isothermal, non-compressible, and non-Newtonian flow. Therefore, three conservation equations were needed. Furthermore, the melt is non-Newtonian, and a constitutive equation was needed.

(1) Conservation equations at the macroscopic level during shearing flow (injection stage)

The mass conservation equation is

(1) ∇·u=0,

the momentum conservation equation is

(2) $$\frac{\partial }{\partial t}\left(\rho u\right)+\nabla \cdot \left(\rho \mathrm{uu}\right)=-\nabla p+\nabla \cdot \tau_{c},$$

and the energy conservation equation is

(3) $$\frac{\partial }{\partial t}\left(\rho c_{p}T\right)+\nabla \cdot \left(\rho c_{p}uT\right)=\nabla \cdot \left(k_{p}\nabla T\right)+\rho \Delta H\frac{\partial \alpha }{\partial t}+\left(-pI+\tau_{c}\right):\nabla u,$$

where ρ is the density; u is the velocity; p is the pressure; $c_{p}$ is the heat capacity; $k_{p}$ is the thermal conductivity; T is the temperature; α is the relative crystallinity; ΔH is the crystallization enthalpy; I is the identity tensor; and $\tau_{c}=\tau_{a}+\tau_{sc}$ is the composite tensor, with $\tau_{a}$ being the stress of the amorphous phase and $\tau_{sc}$ the stress of the semicrystalline phase. 

We used the conception of Zheng et al. [24] to describe the non-Newtonian system. The amorphous phase is described by FENE-P (finite extensible nonlinear elastic model with a Peterlin closure approximation) dumbbells, while the semicrystalline phase is modeled as rigid dumbbells. The stress caused by FENE-P dumbbells is [24]

(4) $$\tau_{a}=nkT\left(\frac{C}{1-trC/b}-I\right),$$

and the evolution of the conformation tensor is [24]

(5) $$\lambda_{\alpha }\left(T\right)\overset{\nabla }{C}+\left[\frac{1}{1-trC/b}C-I\right]=0,$$

where n is the number density of the molecules, k is the Boltzmann constant, b is the dimensionless parameter of the nonlinear spring, tr is the trace of the matrix, C is the configuration tensor, and the upper-convected derivative of C is defined as $\overset{\nabla }{C}=DC/Dt-{\left(\nabla u\right)}^{T}\cdot C-C\cdot \left(\nabla u\right)$. $\lambda_{\alpha }$ is the relaxation time of the fluid, which obeys the Arrhenius equation, namely [24],

(6) $$\lambda_{\alpha }\left(T\right)=\exp\left[\frac{E_{a}}{R_{g}}\left(\frac{1}{T}-\frac{1}{T_{r}}\right)\right]\lambda_{a,r},$$

where $T_{r}$ is a reference temperature, and $E_{a}/R_{g}$ is a constant that can be determined from the experimental data. The stress caused by rigid dumbbells is [24]

(7) $$\tau_{sc}=\frac{\eta_{sc}}{\lambda_{sc}}\left[\langle \mathrm{RR}\rangle +\lambda_{sc}\dot{\gamma }:\langle \mathrm{RRRR}\rangle \right],$$

where $\lambda_{sc}$ is the relaxation time of the rigid dumbbells, $\eta_{sc}$ is the viscosity of the semicrystalline system, $\dot{\gamma }$ is the deformation tensor, 〈RR〉 is the second-order orientation tensor, and 〈RRRR〉 is the fourth-order orientation tensor. The evolution of the orientation tensor 〈RR〉 is defined as [24]

(8) $$\langle \overset{\nabla }{\mathrm{RR}}\rangle =-\frac{1}{\lambda_{sc}}\left(\langle \mathrm{RR}\rangle -\frac{I}{2}-\dot{\gamma }:\langle \mathrm{RRRR}\rangle \right)$$

The relationship between the viscosity of the semicrystalline system and the amorphous system is dependent on the following empirical equation [24]

(9) $$\frac{\eta_{sc}\left(x,T\right)}{\eta_{a}\left(T\right)}=\frac{{\left(\alpha /A\right)}^{\beta_{1}}}{{\left(1-\alpha /A\right)}^{\beta }}\;\alpha <A,$$

and the relaxation times of the rigid dumbbells and FENE-P dumbbells are [24]

(10) $$\frac{\lambda_{sc}\left(x,T\right)}{\lambda_{\alpha }\left(T\right)}=\frac{{\left(\alpha /A\right)}^{\beta_{1}}}{{\left(1-\alpha /A\right)}^{\beta }}\;\alpha <A,$$

where $\beta_{1}$, $\beta_{1}$ and *A* are the empirical constants. Equation (9) clearly shows that when α→A (*A* being the “critical value” of the degree of crystallinity), the viscosity of the semicrystalline system approaches infinity.

To calculate the second-order orientation tensor 〈RR〉 from Equations (7) and (8), one shall use a closure approximation—such as the hybrid [25], EBOF (eigenvalue-based orthotropic fitting) [26,27], or IBOF (invariant-based orthotropic fitting) [28] method—to gain an expression of 〈RRRR〉 in terms of 〈RR〉. Here, the hybrid expression was used, namely,

(11) $${\langle \mathrm{RRRR}\rangle }_{ijkl}={\langle \mathrm{RR}\rangle }_{ij}{\langle \mathrm{RR}\rangle }_{kl},$$

where ${\langle \mathrm{RR}\rangle }_{ij}$ and ${\langle \mathrm{RR}\rangle }_{kl}$ are the components of 〈RR〉, and ${\langle \mathrm{RRRR}\rangle }_{ijkl}$ is the component of 〈RRRR〉.

(2) Conservation equations at the macroscopic level after shearing flow (cooling stage)

In the second case, we assumed there is no fluid flow and the melt is stationary. Therefore, the conservation equation was the energy equation, which can be written as follows,

(12) $$\rho c_{p}\frac{\partial T}{\partial t}=\nabla \cdot \left(k_{p}\nabla T\right)+\rho \Delta H\frac{\partial \alpha }{\partial t}.$$

Actually, for high accuracy, the material parameters can be calculated with the mixture rule. For example, $\rho =\alpha \rho_{sc}+\left(1-\alpha \right)\rho_{\alpha }$, with $\rho_{sc}$ as the density of the semicrystalline phase and $\rho_{\alpha }$ as the density of the amorphous phase.

### 2.2. Crystal Evolution Model at the Microscopic Level

In injection processing, both the spherulitical structure and shish-kebab structure appear in polymer products. The former one is caused by temperature and is known as quiescent crystallization; the latter one is caused by shear or stress and is known as flow-induced crystallization. 

In the morphological simulation, crystals follow the steps of nucleation-growth-impingement. Therefore, it is important to model the nucleation and growth of spherulites and shish-kebabs. In our previous study [21,23], we deduced the evolution equations of spherulites and shish-kebabs based on the Eder model [13] and Schneider rate model [14]. Here, we used the equations obtained in our previous work [23].

We assumed the relationship between the nucleation of spherulites ($N_{s}$) and temperature is [29]

(13) $$N_{s}\left(T\right)=N_{0}\exp\left[a_{n}\Delta T+b_{n}\right],$$

where $a_{n}$ and $b_{n}$ are constants, and $\Delta T=T_{m}^{0}-T$ is the degree of supercooling, with $T_{m}^{0}$ being the equilibrium melting temperature. We shall mention that different nucleation relations of $N_{s}$ have been reported, and the reviews of Pantani et al. [5] are helpful. Usually, these relations may be quite restricting depending on the conditions or materials. 

The growth rate of spherulites ($G_{s}$) is often adopted by the Hoffman–Lauritzen expression [30], which is

(14) $$G_{s}\left(T\right)=G_{0}\exp\left[-\frac{U^{*}}{R_{g}\left(T-T_{\infty }\right)}\right]\exp\left[-\frac{K_{g}}{T\Delta Tf}\right],$$

where $G_{0}$ and $K_{g}$ are constants, $U^{*}$ is the activation energy of motion, $R_{g}$ is the gas constant, $T_{\infty }=T_{g}-30$ (where $T_{g}$ is the glass transition temperature), and $f=2T/\left(T_{m}^{0}+T\right)$.

We assumed the driving force of the nucleation of shish-kebabs ($N_{s-k}$) is the first normal stress difference, which can be written as [29]

(15) $${\dot{N}}_{s-k}=CN_{1},$$

where C is a constant, and $N_{1}$ is the first normal stress difference that can be calculated by Equations (4) and (7). Notice that the driving force for flow-induced nucleation is not well understood, and different approaches have been proposed. Examples of the driving forces include the shear rate [13], shear strain [31], recoverable strain [15], dumbbell free energy [24]. Equation (15) is the simplest but is also widely used in simulations [29,32].

Shish-kebabs are assumed to grow as a cylinder, which means they can grow in two directions, namely, along the length and radial directions. According to Eder [13], the length growth rate ($G_{s-k,l}$) obeys the following equation

(16) $$G_{s-k,l}={\dot{\gamma }}^{2}\cdot g_{1}/{\dot{\gamma }}_{l}^{2},$$

where $g_{1}/{\dot{\gamma }}_{l}^{2}$ is a constant, and $\dot{\gamma }$ is the shear rate. The radial growth rate of the shish-kebabs ($G_{s-k,r}$) is always assumed to be equal to the growth rate of the spherulites, which is

(17) $$G_{s-k,r}=G_{s}.$$

## 3. Multiscale Method

The conception of a multiscale method here is similar to the method we built up in the Couette flow case [22]. We used different methods at different scales and then coupled them together. The finite volume method at the macroscopic level is constructed to calculate the velocity, temperature, stress, etc. The finite volume method is a conservation method. It has advantages of small storage and cheap computational cost, as well as easy handling of the couplings of velocity-pressure, velocity-stress, etc. [33]. Therefore, the finite volume method is widely used in CFD (computational fluid dynamics). The Monte Carlo method at the microscopic level is constructed to capture the development of crystals. The Monte Carlo method is also known as a stochastic simulation and can address the stochastic birth-growth process of spherulites and shish-kebabs. The advantages of the Monte Carlo method are that it can avoid the use of a crystallization kinetics model and it can also predict the detailed morphology evolution. The finite volume method and Monte Carlo method were implemented on different grids—namely, the finite volume method was used on a coarse grid to solve macroscopic Equations (1)–(3), (5), (8), and (12) to obtain the velocity, pressure, stress, and temperature, and the Monte Carlo method was used on a fine grid to capture the evolution of crystals by using Equations (13)–(17) to obtain the relative crystallinity. We refer to our previous work for the arrangement of the coarse grid and fine grid [22,34].

In the modeling part, we put fully coupled mass, momentum, and energy conservation equations together with the constitutive equations of amorphous and semicrystalline phases during the shear treatment (injection stage). However, in the algorithm of the finite volume method, we present a decoupled one. We solved the non-isothermal Newtonian flow to achieve the velocity and temperature. Then, with the results of velocity and temperature, we solved the constitutive equations of amorphous and semicrystalline phases. In other words, the velocity, pressure, and temperature were coupled, while the stress was decoupled. This is because the stresses caused by the amorphous and semicrystalline phases are seriously dependent on the relative crystallinity: a slight increase in relative crystallinity causes a dramatic increase in viscosity, which leads to a large increase in stress. If we put this stress into the momentum equation, we cannot obtain a convergent result because of the large stress source term. Therefore, in our simulation, Equations (1)–(3), (5), and (8) were not solved simultaneously. Actually, when we solve Equations (1)–(3), the SIMPLE method is used [35] . We assumed the flow is a non-isothermal Newtonian flow, which is incompressible. The collocated finite volume method was used. Compared with the finite volume method on a staggered grid, the collocated finite volume method has the advantage of easy implementation on the same grid to overcome the decoupling of velocity-pressure and velocity-stress [33]. It is also noted that in our previous work on the Couette flow model, a continuity equation could not be calculated that avoided the decoupling of velocity-pressure. Detailed implementation of the collocated finite volume method were as follows.

Equations (1)–(3), (5), and (8) can be written as a general transport equation

(18) $$\frac{\partial \left(\delta \varphi \right)}{\partial t}+\nabla \cdot \left(mu\varphi \right)=\nabla \cdot \left(\Gamma \nabla \varphi \right)+S_{\varphi },$$

where δ, m, and Γ are constant, and φ and $S_{\varphi }$ are the functions that are defined in Table 1. The terms in Equation (18) represent the transient, convective, diffusive and source contributions. 

**Figure 3:**
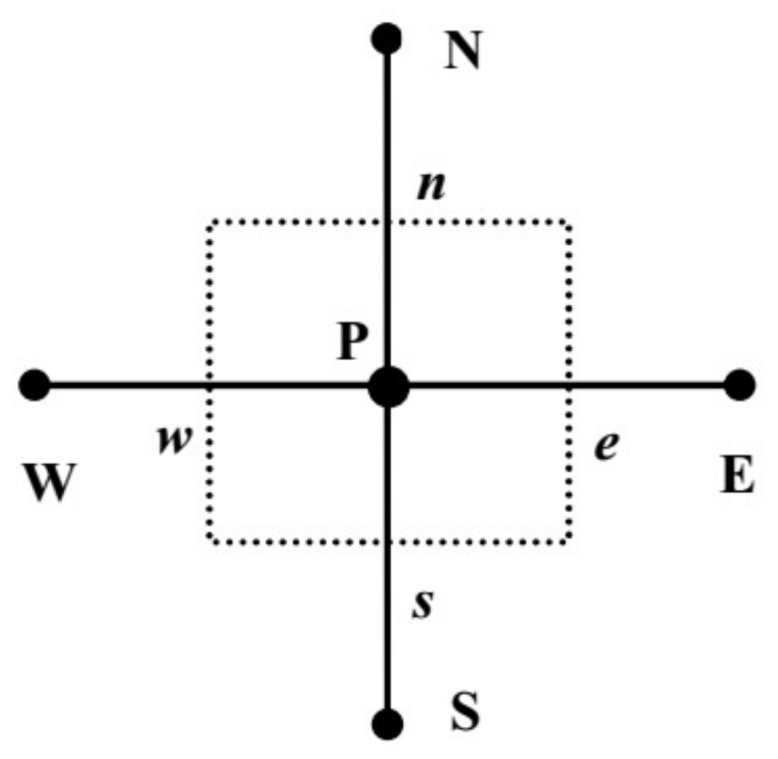
A general control volume.

Equation (18) is integrated over the finite volume cell shown in Figure 3 in space, and the use of the divergence theorem yields

(19) $$\begin{array}{cc} \int_{V}\frac{\partial \left(\delta \varphi \right)}{\partial t}dV+ & \int_{s}^{n}\left[{\left(mu\varphi -\Gamma \nabla \varphi \right)}_{e}-{\left(mu\varphi -\Gamma \nabla \varphi \right)}_{w}\right]dy\\ & +\int_{w}^{e}\left[{\left(mu\varphi -\Gamma \nabla \varphi \right)}_{n}-{\left(mu\varphi -\Gamma \nabla \varphi \right)}_{s}\right]dx=\overset{n}{\underset{s}{\int }}\overset{e}{\underset{w}{\int }}S_{\varphi }dxdy. \end{array}$$

The transient term in Equation (19) is integrated in time and then divided by Δt. A linear approximation is used, which leads to

(20) $$\frac{1}{\Delta t}\int_{t}\delta \frac{\partial \varphi }{\partial t}dVdt\approx \frac{\delta V\left(\varphi_{P}-\varphi_{p}^{0}\right)}{\Delta t},$$

where the superscript “0” indicates the value at the previous time step. The upwind scheme and central differences are used to approximate the convective and diffusive fluxes across each face, respectively. This gives rise to the following discretization

(21) $$A_{P}\phi_{P}=A_{E}\phi_{E}+A_{W}\phi_{W}+A_{N}\phi_{N}+A_{S}\phi_{S}+Q_{P},$$

where $A_{P}$, $A_{E}$, $A_{W}$, $A_{N}$, and $A_{S}$ are the coefficients of $\phi_{P}$, $\phi_{E}$, $\phi_{W}$, $\phi_{N}$, and $\phi_{S}$, respectively, and $Q_{P}$ is the source term. The Gauss–Seidel iteration method can be used to solve the above linear equations. Note that a Rhie–Chow-type [36] interpolation should be used to overcome the pressure-velocity decoupling. Details can be found in the work of Oliveira et al. [37] and Ruan et al. [38].

The Monte Carlo method was employed on the fine grid to track the development of spherulites and shish-kebabs. With the crystal evolution model of Equations (13)–(17), the detailed morphology can be obtained by the Monte Carlo method. This is the main advantage that the morphological simulation has. Furthermore, by using the Monte Carlo method, the relative crystallinity can also be obtained from the volume fraction of crystals under the assumption that the semicrystalline phase is spatially uniform. Thus, the relative crystallinity was calculated by the following equation

*α* = number of cells that are occupied by crystals/total number of cells. (22)

Here, we shall not show the detailed implementation of the Monte Carlo method, but refer to our previous work [21,23] for more details.

**Figure 4:**
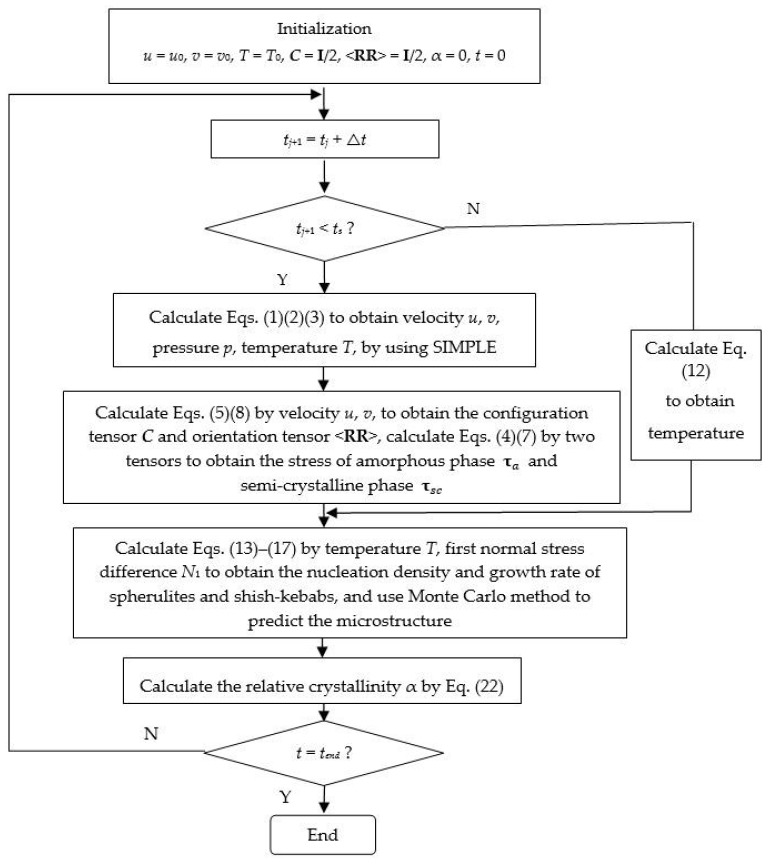
Flowchart for multiscale method in the simulation.

Figure 4 shows the flowchart of the implementation of the multiscale method. 

## 4. Results and Discussion

### 4.1. Problem Definition

Considering the injection mold shown in Figure 2, the length is *L* = 16 mm, and the thickness is *W* = 8 mm. We assumed the walls, with *y* = 0 mm and *y* = 8 mm, experience a constant cooling rate operation and set the boundary conditions as $T=T_{0}-c\times t$, with $T_{0}$ as the initial temperature and *c* as the cooling rate. The other boundary conditions were assumed as ∂T/∂n=0, with n as the unit normal vector. Note that the mold is a thin wall with thickness in the *y* direction. Because of the complexity of the multiscale algorithm, here, we set the length in the *x* direction as twice the length as in the *y* direction. The adiabatic boundary conditions of *x* = 0 mm and *x* = 16 mm were used to obtain a condition similar to the thin-wall thickness of mold in industry. Moreover, here, we set a shear flow to account for the flow and flow history of the injection processing. We assumed the velocity at the inlet is u=Uy(W−y), where U is a constant, and last with the shear time $t_{s}$ s; once the time reaches the “shear time” ($t_{s}$), the flow field is vanished. We fixed the shear time to $t_{s}$ = 10 s and will not discuss this effect later.

The material here was the isotactic polypropylene homopolymer with tacticity 0.96. The parameters used were [5,15,24,29]: $a_{n}$ = 0.156 K/m^3^, $b_{n}$ = 15.1 /m^3^, $G_{0}$ = 2.83 × 10^2^ m/s, $U^{*}/R_{g}$ = 755 K, $K_{g}$ = 5.5 × 10^5^ K^2^, $T_{m}^{0}$ = 483 K, $T_{g}=$ 269 K, $g_{l}/{\dot{\gamma_{l}}}^{2}$ = 2.69 × 10^−7^, C = 10^6^ /Pa/s^2^/m, $\lambda_{\alpha ,r}$ = 4.00 × 10^−2^ s, $T_{r}$ = 476.15 K, $E_{\alpha }/R_{g}$ = 5.602 × 10^3^ K, b = 5, n = 1.26 × 10^26^ /m^3^, k = 1.38 × 10^−23^, β = 9.2, β_1_ = 0.05, and A = 0.44. The other parameters we chose were ρ = 900 kg/m^3^, $c_{p}$ = 2.14 × 10^3^ J/kg/K, *k_p_* = 0.193 W/m/K, ΔH = 107 × 10^3^ J/kg, $T_{0}$ = 490 K, *c* = 2 K/min, and U = 625. In the implementation of the multiscale algorithm, the coarse grid was chosen as 16 × 18, and the fine grid was chosen as 500 × 500.

**Figure 5:**
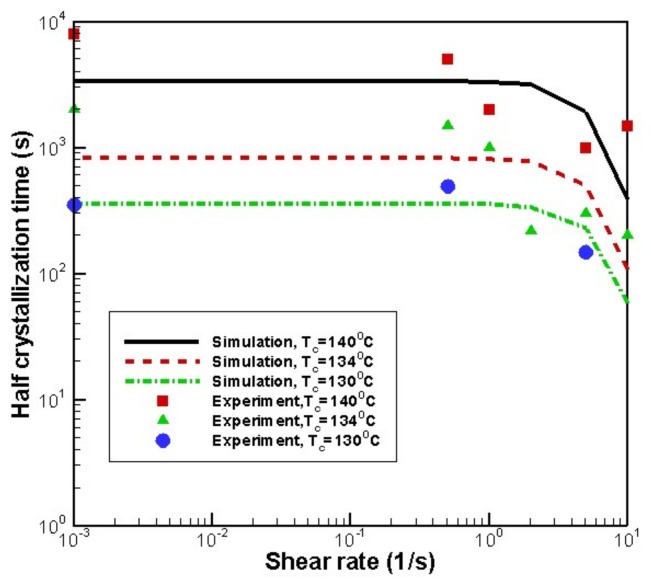
Comparison of simulation result with the experimental result [29].

To show the validity of the model and the Monte Carlo method used at the microscopic level, isothermal crystallization was considered. Figure 5 shows the simulated data with the experimental data [29]. It can be seen that the numerical relationship between the shear rate and the half crystallization time is in good agreement with the experimental data. Therefore, our model and Monte Carlo method is valid.

### 4.2. Temperature, Relative Crystallinity Distribution, and “Skin-Core-Skin” Structure

**Figure 6:**
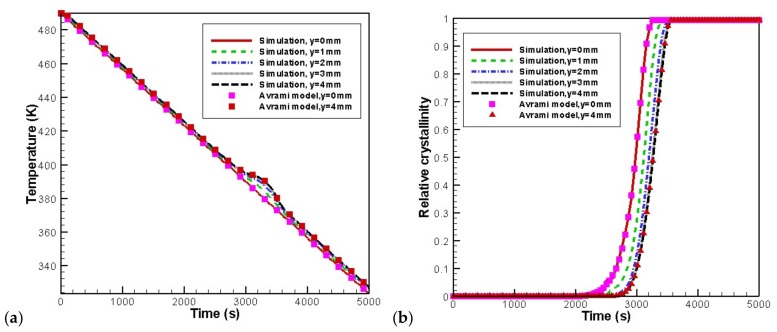
Evolution of temperature and relative crystallinity with time at the profile of *x* = 8 mm: (**a**) temperature, (**b**) relative crystallinity.

Figure 6 shows the temperature and relative crystallinity evolution at the profile of *x* = 8 mm. Results obtained for our algorithm are compared with the Avrami model. Here, the Avrami model is $\alpha =1-\exp\left(-\alpha_{f}\right)$, with $\alpha_{f}=V_{sp}+V_{sh}$, where $V_{sp}$ is the undisturbed total volume of spherulites and $V_{sh}$ is the undisturbed total volume of shish-kebabs. The Schneider rate equation [14,15] was used to compute $V_{sp}$ and the Eder model [13,15] was used to calculate $V_{sh}$. The “Avrami model” in the temperature curves means that the temperature is calculated with the *α* obtained by the Avrami model. As can be seen in Figure 6, the simulation data show a good agreement with the Avrami model. In addition, as shown in the temperature curves, there is a “platform” in the core layer near 2800–3600 s. According to the evolution of relative crystallinity, the relative crystallinity value of the core layer in this period increases rapidly and finally reaches 1. Therefore, this period is the time that crystallization happens. Because of the latent heat released by crystallization, the temperature “platform” forms in the core layer. Furthermore, the crystallization rate in the skin layer is faster than that in the core layer. This result is consistent with our previous study on quiescent crystallization [39].

**Figure 7:**
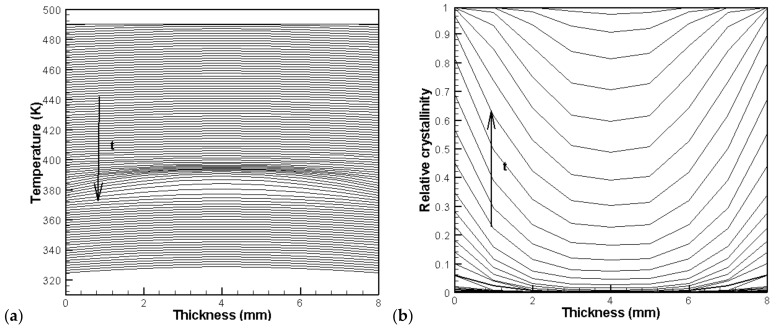
Distribution of temperature and relative crystallinity at different thicknesses: (**a**) temperature, (**b**) relative crystallinity.

Figure 7 shows the evolution of temperature and relative crystallinity at different thicknesses in the profile at *x* = 8 mm. It is evident that crystallization occurs near 400–380 K. It is also clear that crystallization finishes earlier in the skin layer because of the lower temperature at the walls. 

**Figure 8:**
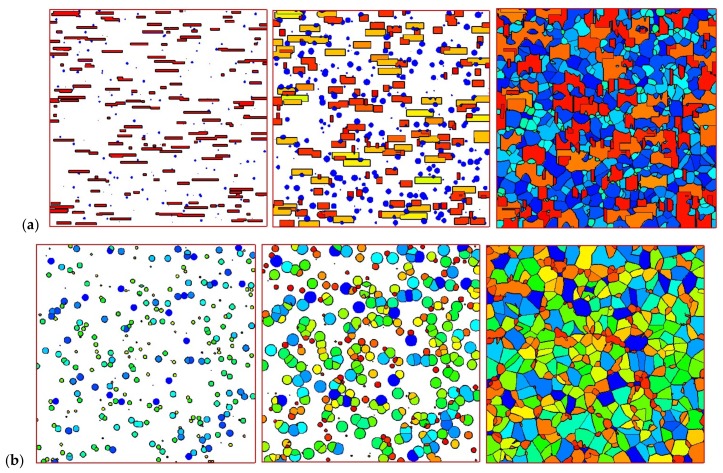
Morphology evolution in the polymer control volume: (**a**) skin volume and (**b**) core volume.

Figure 8 shows the development of crystals in the control volume of the skin layer (8 mm, 0 mm) and in the control volume of the core layer (8 mm, 4 mm). It is clear that in the skin layer, the shish-kebab structure is dominant, while in the core layer, only the spherulitic structure appears. Crystals follow the steps of nucleation, growth, impingement, and, finally, fully filling the whole space. In fact, the shear rate near the skin layer is large, which is of benefit to the nucleation and growth of shish-kebabs. However, the shear rate is often absent or small in the core layer, which is not suitable to the development of shish-kebabs. However, a lower temperature is favorable for the nucleation and growth of spherulites. This development of crystal morphology is in agreement with the experimental finding of Koscher et al. [29].

**Figure 9:**
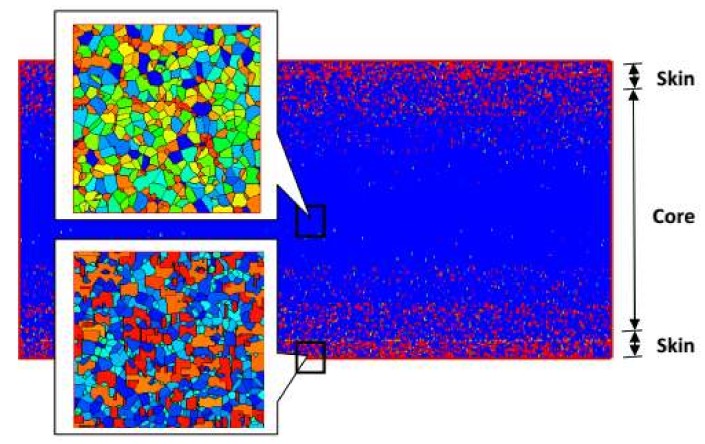
“Skin-core-skin” structure.

Figure 9 shows the final crystal morphology in the computational domain. The structure takes on a typical “skin-core-skin” structure: in the skin layer, the crystal structure is mainly composed of the anisotropic shish-kebab; in the core layer, the crystal structure is the isotropic spherulite. This structure is consistent with experimental findings [5,40,41,42]. Our approach is valid in revealing the crystal microstructures. It should be mentioned that in our simulation, we do not consider a “frozen layer” [5,40,41,42]. Here, we consider a moderate temperature cooling boundary condition; therefore, a “frozen layer” cannot be captured in this case.

**Figure 10:**
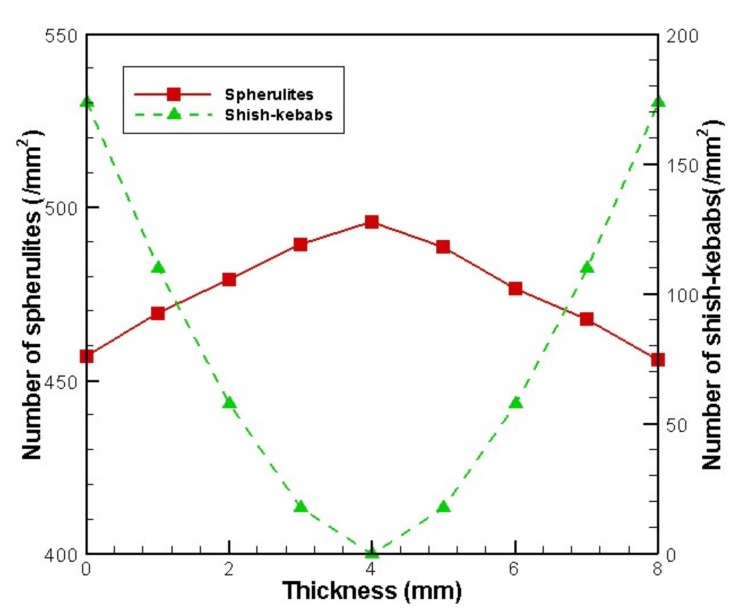
Number of spherulites and shish-kebabs at the different thicknesses at the profile of *x* = 8 mm.

The Monte Carlo simulation also allowed us to show the details of spherulites and shish-kebabs. Figure 10 shows the number of spherulites and shish-kebabs at different thicknesses at the profile of *x* = 8 mm. It is evident that the number of shish-kebabs decreases from the skin to core, while the number of spherulites increases from the skin to core. The trend in number of shish-kebabs is caused by the change in shear rate. It is worth noting that the trend in the number of spherulites is in contrast to the quiescent case [40]. In the quiescent case, because of the highest cooling rate being in the skin layer, the number of spherulites is largest, which leads to the smallest size of spherulites. Although in this case, the temperature performance is similar to the quiescent case, the shish-kebab structure appears, which restricts the number of spherulites.

### 4.3. Effects of Temperature Cooling Rate of the Mold Wall

Three cases of temperature cooling rate of the mold wall were examined, namely, *c* = 1 K/min, *c* = 2 K/min, and *c* = 5 K/min. It is noted that the high cooling rate is related to the low wall temperature in real injection processing.

**Figure 11:**
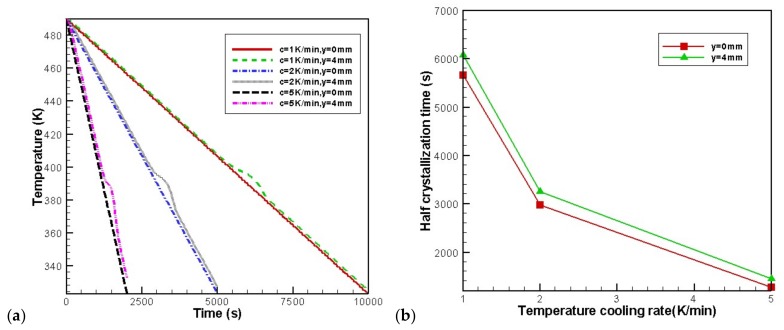
Effect of temperature cooling rate of the mold wall on the temperature and the rates of crystallization at the profile of *x* = 8 mm: (**a**) temperature, (**b**) half crystallization time.

Figure 11 shows the temperature evolution and the rates of crystallization at the profile of *x* = 8 mm. To show the difference between the skin and core layers, we chose the skin point (8 mm, 0 mm) and core point (8 mm, 4 mm) as examples. As seen in Figure 11, the case with a high cooling rate leads to the fast decrease in temperature and high crystallization rate. 

Table 2 shows the parameters related to the microstructure. The average diameter of spherulites, number of shish-kebabs and the relative crystallinity caused by spherulites and shish-kebabs are displayed. It is evident that in the case of the higher cooling rate, the average size of spherulites decreases. However, the number of shish-kebabs does not change with the cooling rate. Therefore, it can be concluded that the temperature cooling rate of the wall mainly affects the nucleation and growth of spherulites. The predicted average diameter of spherulites also shows agreement with the experimental and numerical works of Pantanin et al. [5].

### 4.4. Effects of Initial Melt Temperature

**Figure 12:**
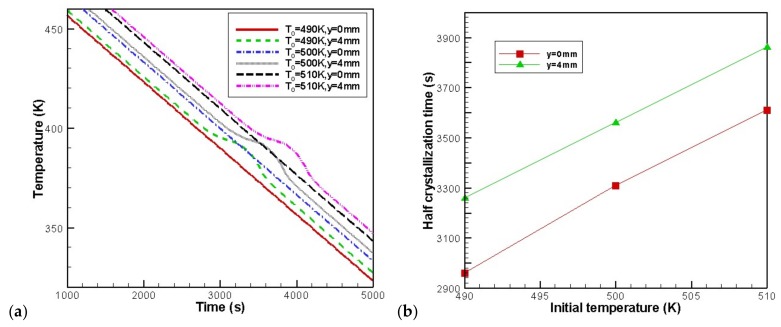
Effect of the initial melt temperature on the temperature and the rates of crystallization at the profile *x* = 8 mm: (**a**) temperature, (**b**) half crystallization time.

Three cases of initial melt temperature were examined, namely, $T_{0}$ = 490 K, $T_{0}$ = 500 K, $T_{0}$ = 510 K. Figure 12 presents the evolution of the temperature and the rates of crystallization at the skin point (8 mm, 0 mm) and core point (8 mm, 4 mm) for different initial melt temperatures. It can be seen from Figure 12 that the higher the initial melt temperature, the later the occurrence of the temperature “platform” and crystallization. However, the curves only shift to the right when the initial melt temperature is increased. 

Table 3 shows the effects of the initial melt temperature on the microstructures. These effects are reflected by the average diameter of spherulites, number of shish-kebabs, and the relative crystallinity contributed by the spherulites and shish-kebabs at the skin and core volume. It is clear that when the initial melt temperature increases, both the contribution and the number of shish-kebabs are reduced. This is because the higher melt temperature reduces the first normal stress difference. According to Equation (15), the number of shish-kebabs reduces, which leads to a reduction in the contribution of shish-kebabs to the relative crystallinity. It is also evident that the initial melt temperature has minor effects on spherulites. 

### 4.5. Effects of the Maximum Velocity of Melt at the Inlet

**Figure 13:**
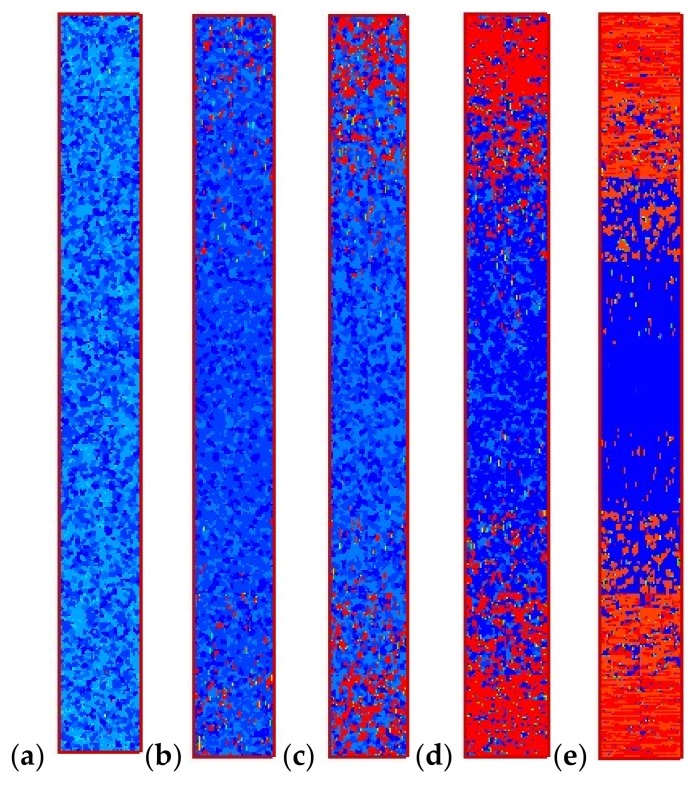
Final crystal morphology with different maximum velocities at the inlet. (**a**) ${\dot{\gamma }}_{\max}$ = 1 s^−1^, (**b**) ${\dot{\gamma }}_{\max}$ = 3 s^−1^, (**c**) ${\dot{\gamma }}_{\max}$ = 5 s^−1^, (**d**) ${\dot{\gamma }}_{\max}$ = 7 s^−1^, (**e**) ${\dot{\gamma }}_{\max}$ = 10 s^−1^.

The effects of maximum velocity at the inlet were also investigated. We changed U from 125 to 1250 to obtain the velocity at the inlet. The velocity also affects the maximum shear rate. The shear rate was calculated with the velocity as $\dot{\gamma }=\left|\partial u/\partial y\right|$. Figure 13 shows the final crystal morphology of the control volume at *x* = 8 mm. When the maximum velocity is small (small shear rate), the shish-kebab structure in the skin layer is not apparent. With the increase in velocity (or shear rate), the shish-kebab structure becomes clear, and the thickness of the skin layer becomes wide. Hence, the velocity at the inlet has significant effects on the crystal morphology.

**Figure 14:**
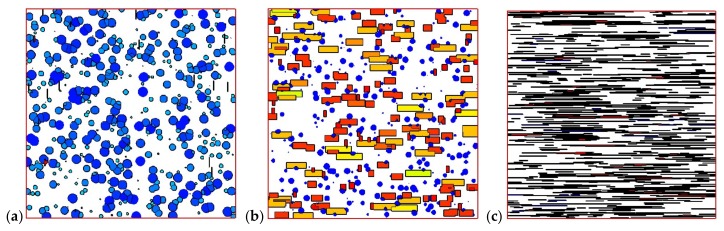
Morphology of the polymer skin volume: (**a**) ${\dot{\gamma }}_{\max}$ = 1 s^−1^, (**b**) ${\dot{\gamma }}_{\max}$ = 5 s^−1^, (**c**) ${\dot{\gamma }}_{\max}$ = 10 s^−1^.

**Figure 15:**
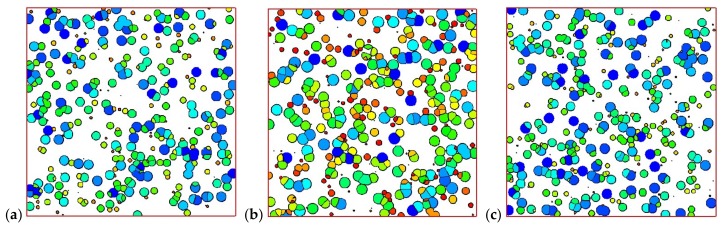
Morphology at the polymer core volume: (**a**) ${\dot{\gamma }}_{\max}$ = 1 s^−1^, (**b**) ${\dot{\gamma }}_{\max}$ = 5 s^−1^, (**c**) ${\dot{\gamma }}_{\max}$ = 10 s^−1^.

We now restrict our attention to the control volume of skin point (8 mm, 0 mm) and core point (8 mm, 4 mm) with different velocities (shear rate). Figure 14 and Figure 15 show the morphologies of the skin and core volumes. As we can see in Figure 14, the morphology of the skin volume changes clearly when the velocity is increased. The number and anisotropy of shish-kebabs become higher in the larger shear rate case of the skin volume. The crystal morphology of the core volume in Figure 15 shows that shear rate has a minor effect on the number and size of spherulites.

Table 4 shows the average diameter of spherulites, number of shish-kebabs, and the relative crystallinity caused by spherulites and shish-kebabs at the skin and core volume. The number of shish-kebabs and the contribution of shish-kebabs to relative crystallinity decrease from the skin to core. Furthermore, the number of shish-kebabs and the contribution of shish-kebabs to the relative crystallinity increase with the maximum velocity, and the impact is significant.

## 5. Conclusions

We have extended the multiscale simulation for polymer crystallization in a pipe flow related to simplified injection processing. The “skin-core-skin” crystal structure was obtained. Both the spherulitical structure and shish-kebab structure can be captured by our algorithm. We have also shown the effects of the mold temperature cooling rate, initial melt temperature, and the maximum velocity of the melt at the inlet on microstructures. The results indicate that the temperature cooling rate of the mold (or mold temperature) especially affects the morphology of spherulites, whereas the initial melt temperature and maximum velocity of the melt at the inlet mainly affect the morphology of the shish-kebabs. We hope the results presented here can provide more insight into the microstructural details of crystallization and thus be more helpful to industrial applications. 

In this work, we did not consider the changing flow domain in the injection stage, and used the viscoelastic flow without a free surface as the flow field for simplicity. To model the crystallization more accurately, a melt with the free surface should be taken into account. Moreover, the temperature boundary condition used here reflects a moderate cooling rate. In real injection processing, a low mold temperature that generates a higher temperature gradient should be applied. Our future work will be concentrated on improving our multiscale method and combining it with other software for simulating real injection processing.

## Figures and Tables

**Table 1:** Definition of constants and functions in the general equation.

Equation	δ	m	ϕ	Γ	$S_{\phi }$
Continuity	0	1	1	0	0
Momentum	ρ	ρ	u	η	−∇p
Energy	$\rho c_{p}$	$\rho c_{p}$	*T*	k	$\rho \Delta H\frac{\partial \alpha }{\partial t}$
FENE-P model	1	1	**C**	0	$-\frac{1}{\lambda_{\alpha }\left(T\right)}\left[\frac{C}{1-\frac{trC}{b}}-I\right]+{\left(\nabla u\right)}^{T}\cdot C+C\cdot \nabla u$
Rigid dumbbell model	1	1	〈RR〉	0	$-\frac{1}{\lambda_{sc}\left(T\right)}\left[\langle \mathrm{RR}\rangle -\frac{I}{2}\right]-\dot{\gamma }:\langle \mathrm{RRRR}\rangle +{\left(\nabla u\right)}^{T}\langle \mathrm{RR}\rangle +\langle \mathrm{RR}\rangle \cdot \nabla u$

**Table 2:** Effect of the temperature cooling rate of the mold wall on the spherulites and shish-kebabs at the profile of *x* = 8 mm.

Thickness, Cooling Rate	Average Diameter of Spherulites (µm)	Relative Crystallinity Contributed by Spherulites	Number of Shish-Kebabs (/mm^2^)	Relative Crystallinity Contributed by Shish-Kebabs
*y* = 0 mm, *c* = 1 K/min	48.63	52.93%	174	47.07%
*y* = 0 mm, *c* = 2 K/min	40.58	57.82%	174	42.18%
*y* = 0 mm, *c* = 5 K/min	29.39	65.53%	176	34.47%
*y* = 4 mm, *c* = 1 K/min	63.06	100%	0	0%
*y* = 4 mm, *c* = 2 K/min	50.66	100%	0	0%
*y* = 4 mm, *c* = 5 K/min	37.63	100%	0	0%

**Table 3:** Effect of initial melt temperature on spherulites and shish-kebabs at the profile *x* = 8 mm.

Thickness, Initial Melt Temperature	Average Diameter of Spherulites (µm)	Relative Crystallinity Contributed by Spherulites	NUMBER of Shish-Kebabs (/mm^2^)	Relative Crystallinity Contributed by Shish-Kebabs
*y* = 0 mm, *T*_0_ = 490 K	40.58	57.82%	174	42.18%
*y* = 0 mm, *T*_0_ = 500 K	39.83	60.77%	140	39.23%
*y* = 0 mm, *T*_0_ = 510 K	40.32	68.15%	112	31.85%
*y* = 4 mm, *T*_0_ = 490 K	50.66	100%	0	0%
*y* = 4 mm, *T*_0_ = 500 K	51.77	100%	0	0%
*y* = 4 mm, *T*_0_ = 510 K	50.53	100%	0	0%

**Table 4:** Effect of the maximum velocity at the inlet on spherulites and shish-kebabs at the profile of *x* = 8 mm.

Thickness, Shear Rate	Average Diameter of Spherulites (µm)	Relative Crystallinity Contributed by Spherulites	Number of Shish-Kebabs (/mm^2^)	Relative Crystallinity Contributed by Shish-Kebabs
*y* = 0 mm, $\dot{\gamma }$ = 1 s^−1^	45.05	98.64%	16	1.36%
*y* = 0 mm, $\dot{\gamma }$ = 5 s^−1^	40.58	57.82%	174	42.18%
*y* = 0 mm, $\dot{\gamma }$ = 10 s^−1^	16.63	3.09%	722	96.91%
*y* = 4 mm, $\dot{\gamma }$ = 1 s^−1^	49.98	100%	0	0%
*y* = 4 mm, $\dot{\gamma }$ = 5 s^−1^	50.66	100%	0	0%
*y* = 4 mm, $\dot{\gamma }$ = 10 s^−1^	49.66	100%	0	0%
